# Financial protection effects of modification of China’s New Cooperative Medical Scheme on rural households with chronic diseases

**DOI:** 10.1186/1472-6963-14-305

**Published:** 2014-07-15

**Authors:** Jing Wang, Lina Chen, Ting Ye, Zhiguo Zhang, Jingdong Ma

**Affiliations:** 1 Department of Health Management, School of Medicine and Health Management, Tongji Medical College, Huazhong University of Science and Technology, Hubei 430030, China; 2 The Key Research Institute of Humanities and Social Science of Hubei Province, Huazhong University of Science and Technology, Hubei 430030, China; 3 Department of Health Information, School of Medicine and Health Management, Tongji Medical College, Huazhong University of Science and Technology, Hubei 430030, China

**Keywords:** Rural China, Health insurance, New Cooperative Medical Scheme, Financial burden, Chronic diseases, Financial protection

## Abstract

**Background:**

Several years have passed since the rural New Cooperative Medical Scheme (NCMS) in China was established and policies kept continuous improvement. Its policies on chronic diseases vary by county but have certain shared characteristics. Following this modification of medical insurance policy, this study reassesses the provision of insurance against expenditure on chronic diseases in rural areas, and analyzes its effect on impoverishment.

**Methods:**

We conducted an empirical study using multi-stage stratified random sampling. We surveyed 1,661 rural households in three provinces and analyzed the responses from 1,525 households that participated in NCMS, using descriptive and logistic regression analysis.

**Results:**

The NCMS has reduced the prevalence of poverty and catastrophic health expenditure (CHE), as measured by out-of-pocket (OOP) payments exceeding 40% of total household expenditure, by decreasing medical expenditure. It provides obvious protection to households which include someone with chronic diseases. However, these households continue to face a higher financial risk than those without anyone suffering from chronic diseases. Variables about health service utilization and OOP payment differed significantly between households with or without people suffering from chronic disease. And CHE risk is commonly associated with household income, the number of family members with chronic diseases, OOP payment of outpatient and inpatient service in all three provinces.

**Conclusion:**

To reduce CHE risk for these households, it is critical to decrease OOP payments for health services by enhancing the effective reimbursement level of NCMS and strictly regulating the providers’ behaviors. We recommend that a combinatory changes should be made to the rural health insurance scheme in China to improve its effect. These include improving the NCMS benefit package by broadening the catalogue of drugs and treatments covered, decreasing or abolishing deductible and increasing the reimbursement ratio of outpatient services for people with chronic diseases, together with expansion of insurance fund, and modifying health providers’ behaviors by payment reform.

## Background

### Financial burden of chronic diseases

The global financial burden from chronic diseases continues to grow rapidly. According to a WHO report in 2009 [1], two out of three deaths each year were owed to chronic diseases, four-fifths of them occurred in low- and middle-income countries, and a third involved people under 60 years of age. Chronic diseases are also related to increasing out-of-pocket (OOP) payments and catastrophic health expenditure (CHE), especially for those with lower incomes. Su et al. found chronic diseases to be one of the key determinants of CHE [2]. Abegunde et al. demonstrated that chronic diseases decreased household welfare, not only because of causing higher levels of household healthcare expenditure, but also productivity losses which could reduce labor supply and therefore household labor income [3].

China, the largest developing country in the world, undoubtedly also faces a growing financial burden from chronic diseases. The fourth National Health Service Survey (NHSS) in 2008 showed the prevalence of chronic disease in sample populations in rural areas was 17.1%, 4.94% higher than that found by the third NHSS in 2003. About 82% of deaths and 70% of Disability-Adjusted Life Years resulted from chronic diseases [4]. Evidence from Sun et al. showed that expenditure on chronic diseases accounted for, on average, 27% of annual non-food per capita expenditure amongst New Cooperative Medical Scheme (NCMS) members in Shandong province, and 35% in Ningxia Hui Autonomous Region [5]. Jiang et al. analyzed the fourth NHSS data and found that prevalence of poverty in households with at least one family member suffering from chronic diseases was more than twice as those without a chronic disease member [6]. The financial burden of chronic diseases in China is expected to increase significantly in the foreseeable future, with population aging, anticipated changes in social and economic structures, and progressive pollution in the environment [7].

### Health insurance protection for the financial burden of chronic diseases

Bankruptcy and poverty following illness has been well documented among people who lack health care insurance in the United States [8], China [9], Korea [10] and Russia [3]. Many countries have established universal health insurance systems to deal with the financial burden of disease. For example, Thailand provides universal health insurance to successfully reduce catastrophic and impoverishing health expenditure [11].

However, the financial protection against chronic diseases provided by medical insurance varies. For example, although Australia has a well-funded universal health insurance system, rising co-payments for medications and private medical consultations, inadequate subsidies for health support, and poor eligibility barriers for existing social support made chronic illness management economically stressful, especially for those with low incomes [12]. Another study showed that a wide segment of the US population, particularly poor families and people with multiple chronic diseases, lacked financial protection to ensure their access to health services [13]. Abegunde et al. found that households frequently depended on informal coping mechanisms in the face of chronic diseases, irrespective of insurance cover [3]. These problems suggest that health insurance systems need continuous modification to provide better financial protection for those with chronic diseases.

### The rural health insurance scheme in China and its effects on financial protection against expenditure on chronic diseases

The main form of rural health insurance in China is NCMS, which was established in 2003. Funding was provided both by residents of rural areas and the central and local government. Local residents decide whether they wish to participate or not. Funding and reimbursement guidelines are issued by the central government and the design and benefit packages vary from county to county, because of different economic conditions and other factors such as the prevalence of certain diseases.

In 2003, the annual premium per capita for the NCMS was very small, only 30 Chinese Yuan (¥) (US$3.62 based on the exchange rate in January, 2003). In most counties, the scheme initially focused on providing insurance against “catastrophic” inpatient expenses, and offered only very limited funding for other expenses.

The goal of the scheme was to protect households against impoverishment caused by medical expenses. However, under the catastrophic coverage model, patients with chronic diseases were often not reimbursed despite incurring high medical expenditures. For example, 54% of medical expenditure on chronic diseases was spent on outpatient services and so was not covered by the NCMS [14]. A study by Shi et al. indicated that the NCMS did not protect against financial catastrophe and household impoverishment owing to health expenditure, especially for the poor and chronically-ill [15]. In 2006 and 2008, Jing et al. conducted household surveys in Shandong and Ningxia in China and found that NCMS reimbursement policies for chronic diseases did not have statistically significant effects on CHE [16]. This confirmed that the NCMS was not achieving its purpose.

By 2008, the NCMS had achieved high coverage of the rural population (over 90%), and provision of protection against the financial burden associated with chronic diseases was regarded as one of its main goals. It began to cover outpatient expenditure from 2009, with more policies available for patients with chronic diseases. The annual premium per capita has gradually increased to ¥340 (US$54.75 based on the exchange rate in January, 2013) in 2013.

Although NCMS policies on chronic diseases vary by county, largely depending on the local pooling arrangements and the prevalence of chronic diseases, there are certain shared characteristics. The NCMS generally covers outpatient expenditure, but the reimbursement ratio for designated chronic diseases is higher than for other illnesses. In some counties, the reimbursement ratio is slightly higher than for ordinary outpatients (about 50%), but still lower than for inpatients. In others, the reimbursement ratio for outpatients with designated chronic diseases is the same as for inpatients, which is about 95% and varies between different levels of hospitals. In most places, outpatient reimbursement has a deductible or a ceiling, but the ceiling is higher for designated chronic diseases than for other diseases. The designated chronic diseases vary by county, but the NCMS covers the most common ones in each county. And the reimbursement eligibility of patients with chronic diseases may need to be confirmed in advance by the local NCMS administration.

It is worth examining whether the implementation of modified NCMS policies for chronic diseases has achieved their aim, by providing better financial protection for rural households. In 2011, we therefore conducted an empirical study by household survey in Zhejiang, Hubei and Chongqing provinces to reassess rural health insurance protection for those with chronic diseases.

## Methods

### Sampling

We selected three provinces for the field survey, Zhejiang, Hubei and Chongqing, from the Eastern, Central and Western regions of China, respectively. Using multi-stage stratified random sampling, three counties were selected from each province or municipality to represent high, medium and low income levels. Two towns were then selected randomly from each county, two villages from each town, and 50 households from each village. Questionnaires were sent to 1,800 households, and 1,661 were returned in a usable state, after eliminating invalid responses (such as those that were incomplete or contained discrepant data). Since this study focuses on the results of the NCMS, we only analyzed responses from the 1,525 households that participated in NCMS.

We used the similar questionnaire to the 2008 fourth NHSS, but with slight modification. The revised questionnaire collected information on household socioeconomic characteristics, income and expenditure, number of family members with chronic illnesses, healthcare utilization and healthcare expenditure.

### Definition of some concepts

A person with chronic diseases in this study is defined as an individual reported to have been diagnosed with diabetes, hypertension, heart disease, malignant tumor, rheumatic arthritis or chronic obstructive pulmonary disease before the survey. These six conditions are covered by most counties’ NCMS chronic reimbursement schemes. And they may cover the vast majority of people with chronic diseases in rural China [6].

Household’s health expenditures include the total payments of each family member for outpatient care, inpatient care, self-prescribed medication and traditional Chinese medicine. OOP payments are self-reported payments for co-insurance, co-payments, deductibles, and other related medicines, items and services not covered by medical insurance. The recall period of outpatient care was 2 weeks preceding the survey, and the recall period of inpatient care was 1 year. Since this study focused on measuring the financial burden directly related to medical insurance, insurance premiums were excluded.

CHE is defined as OOP payments exceeding a certain threshold in a given period. Although various thresholds have been used in previous studies, we used that of Kawabata and Xu et al. [17,18], OOP payments exceeding 40% of total household expenditure.

Households impoverished by medical expenditure are those whose expenditure per capita was originally above the poverty line, but fell below that line because of medical expenditure. The poverty line used in this study was a net annual per capita income of less than 2,300 RMB (US$347.43, based on the exchange rate in January, 2011). This is the national poverty line defined by the Chinese government in 2011.

### Analyses

To highlight the differences between households with and without people suffering from chronic diseases, we compared the variables related to household socioeconomic characteristics and health service utilization in three provinces. The variable definitions and their abbreviations are set out in Table 1. The variables included continuous and categorical variables. Continuous variables were analyzed using One-way ANOVA to compare difference of means and difference in proportions, while categorical variables were analyzed by a Chi-square test.

**Table 1:** Definitions and abbreviations of variables

**Variable**	**Definition**	**Abbreviation**	**Variable type**
Household size	Number of household members	H-size	continuous
Household with aged members	Household contains someone aged 60 years or over	H-aged	categorical
Highest education	The highest level of education of any household member (primary school level or below, higher level of education).	H-edu	categorical
Household income level	Household income quintiles	H-inco	categorical
Household employment status	Household head’s employment status	H-emp	categorical
Poverty	Whether the household above or below poverty line	Pov	categorical
Outpatient utilization	Number of times that household members have received outpatient services in the last 14 days	Op-uti	continuous
OOP of outpatient	OOP payments for outpatient services made by the household in the last 14 days	Op-OOP	continuous
Inpatient utilization	Number of times in the last year that household members have used inpatient services	Ip-uti	continuous
OOP of inpatient	OOP payments for inpatient services made by the household in the last year	Ip-OOP	continuous
Household members with chronic diseases	Number of people in the household with chronic diseases	H-chro	continuous

We then applied logistic regression analysis to identify the relationship between variables and CHE after NCMS reimbursement (1, with CHE; 0, without CHE), with algorithms of backward steps. To describe the effect of NCMS, we calculated the prevalence of poverty and CHE before and after NCMS reimbursement, and compared the situation for households with and without people suffering from chronic diseases in the three provinces.

All data were coded and computerized using EpiData 3.0 software and analyzed with SPSS 12.0 software. A p-value of less than or equal to 0.05 was considered statistically significant.

This study was approved by the Ethics Committee of Tongji Medical College, Huazhong University of Science and Technology.

## Results

Table 2 shows differences in variables between households with and without people suffering from chronic diseases. Five variables, including H-aged, H-edu, Op-uti, Op-OOP and Ip-uti, differed significantly between these types of households in all three provinces. Households with people suffering from chronic diseases had more elder members, a higher ratio of lower educational levels, more outpatient visits, higher OOP payments for outpatient services in the previous 14 days, and utilized more inpatient services in the last year than those without people suffering from chronic diseases. There were also some variables with significant differences in particular provinces between those households with and without people suffering from chronic disease: H-size in Chongqing, H-inco in both Hubei and Chongqing, Pov in Hubei, and Ip-OOP in both Chongqing and Zhejiang.

**Table 2:** Comparison of variables between households with and without members suffering from chronic diseases

	**Hubei**			**Chongqing**			**Zhejiang**		
	**Households with members suffering from chronic diseases**	**Households without chronic diseases**	p-value*	**Households with members suffering from chronic diseases**	**Households without chronic diseases**	p-value*	**Households with members suffering from chronic diseases**	**Households without chronic diseases**	p-value*
Mean of H-size (member)	3.83	3.88	p = 0.663	3.91	4.29	p = 0.020	3.85	4.04	p = 0.305
H-aged (N (% within province))									
→ Yes	179 (28.28%)	171 (27.01%)	p = 0.006	186 (37.35%)	132 (26.51%)	p < 0.001	113 (28.68%)	122 (30.96%)	p < 0.001
→ No	114 (18.01%)	169 (26.70%)		64 (12.85%)	116 (23.29%)		30 (7.61%)	129 (32.74%)	
H-edu (N (% within province))									
→ Primary school or less	68 (10.74%)	53 (8.37%)	p = 0.015	78 (15.66%)	52 (10.44%)	p = 0.009	49 (12.44%)	62 (15.74%)	p = 0.042
→ Higher level of education	225 (35.55%)	287 (45.34%)		172 (34.54%)	196 (39.36%)		94 (23.86%)	189 (47.97%)	
H-income (N (% within province))									
→ Very poor	123 (19.43%)	118 (18.64%)	p = 0.028	97 (19.48%)	58 (11.65%)	p = 0.002	38 (9.64%)	41 (10.41%)	p = 0.110
→ Poor	73 (11.53%)	96 (15.17%)		89 (17.87%)	95 (19.08%)		29 (7.36%)	53 (13.45%)	
→ Middle	69 (10.90%)	67 (10.58%)		39 (7.83%)	49 (9.84%)		31 (7.87%)	54 (13.71%)	
→ Rich	19 (3.00%)	41 (6.48%)		11 (2.21%)	22 (4.42%)		25 (6.35%)	64 (16.24%)	
→ Very rich	9 (1.42%)	18 (2.84%)		14 (2.81%)	24 (4.82%)		20 (5.08%)	39 (9.90%)	
H-emp (N (% within province))									
→ Employee	13 (2.05%)	22 (3.48%)	p = 0.312	8 (1.61%)	9 (1.81%)	p = 0.049	6 (1.52%)	25 (6.35%)	p = 0.076
→ Farmer	223 (35.23%)	242 (38.23%)		188 (37.75%)	162 (32.53%)		79 (20.05%)	142 (36.04%)	
→ Casual worker	57 (9.00%)	76 (12.01%)		54 (10.84%)	77 (15.46%)		58 (14.72%)	84 (21.32%)	
Pov (N (% within province))									
→ Under poverty line	72 (11.37%)	56 (8.85%)	p = 0.011	46 (9.24%)	34 (6.83%)	p = 0.154	15 (3.81%)	19 (4.82%)	p = 0.321
→ Above poverty line	221 (34.91%)	284 (44.87%)		204 (40.96%)	214 (42.97%)		128 (32.49%)	232 (58.88%)	
Mean of Op-uti (times)	0.65	0.17	p < 0.001	0.41	0.15	p < 0.001	0.24	0.13	p = 0.027
Mean of Op-OOP (yuan)	344.92	53.51	p < 0.001	185.31	33.04	p < 0.001	167.64	64.02	p = 0.010
Mean of Ip-uti (times)	0.58	0.21	p < 0.001	0.53	0.32	p < 0.001	0.40	0.23	p = 0.003
Mean of Ip-OOP (yuan)	3407.24	1897.36	p = 0.148	2454.98	1044.38	p = 0.007	3798.60	1048.80	p = 0.003

*α = 0.05.

Table 3 illustrates the results of the logistic regression analysis to explain the relationship between variables and CHE after NCMS reimbursement. Four variables, including H-inco, H-chro, Op-OOP and Ip-OOP, were all included in the logistic regression equation of the three provinces. Only H-inco was a protection factor for CHE, which unsurprisingly means likelihood of CHE decreases with increasing household income. Ip-uti in Chongqing and Zhejiang, and H-size in Zhejiang could also affect CHE.

**Table 3:** Logistic regression: relationships between variables and CHE after NCMS reimbursement

		**B**	**S.E.**	**Wald**	**df**	**p-value**	**Exp (B)**	**95% C.I. for EXP(B)**	
								**Lower**	**Upper**
Hubei*	H-income^#^			33.119	4	0.000			
	→ Poor	-0.713	0.348	4.207	1	0.040	0.490	0.248	0.969
	→ Middle	-2.375	0.533	19.865	1	0.000	0.093	0.033	0.264
	→ Rich	-2.808	0.826	11.566	1	0.001	0.060	0.012	0.304
	→ Very rich	-7.339	1.628	20.328	1	0.000	0.001	0.000	0.016
	H-chro	0.850	0.210	16.391	1	0.000	2.339	1.550	3.529
	Op-OOP	0.015	0.002	58.214	1	0.000	1.015	1.011	1.019
	Ip-OOP	0.001	0.000	50.431	1	0.000	1.001	1.000	1.001
	Constant	-2.378	0.266	79.885	1	0.000	0.093		
Chongqing**	H-income^#^			40.500	4	0.000			
	→ Poor	-1.759	0.370	22.546	1	0.000	0.172	0.083	0.356
	→ Middle	-1.982	0.540	13.486	1	0.000	0.138	0.048	0.397
	→ Rich	-3.865	1.134	11.618	1	0.001	0.021	0.002	0.194
	→ Very rich	-7.955	2.148	13.709	1	0.000	0.000	0.000	0.024
	H-chro	1.138	0.269	17.891	1	0.000	3.120	1.842	5.286
	Op-OOP	0.012	0.002	42.400	1	0.000	1.012	1.008	1.016
	Ip-uti	0.942	0.329	8.227	1	0.004	2.566	1.348	4.886
	Ip-OOP	0.000	0.000	13.304	1	0.000	1.000	1.000	1.001
	Constant	-2.300	0.322	51.045	1	0.000	0.100		
Zhejiang***	H-size	-0.415	0.169	6.023	1	0.014	0.660	0.474	0.920
	H-income^#^			11.202	4	0.024			
	→ Poor	-0.779	0.659	1.399	1	0.237	0.459	0.126	1.668
	→ Middle	-1.185	0.759	2.438	1	0.118	0.306	0.069	1.353
	→ Rich	-2.355	0.918	6.577	1	0.010	0.095	0.016	0.574
	→ Very rich	-3.126	1.075	8.456	1	0.004	0.044	0.005	0.361
	H-chro	1.282	0.307	17.425	1	0.000	3.603	1.974	6.578
	Op-OOP	0.009	0.001	37.985	1	0.000	1.009	1.006	1.012
	Ip-uti	1.711	0.488	12.298	1	0.000	5.532	2.127	14.390
	Ip-OOP	0.000	0.000	7.753	1	0.005	1.000	1.000	1.000
	Constant	-1.982	0.545	13.211	1	0.000	0.138		

* -2 Log likelihood is 307.754, Cox & Snell R Square is 0.529, and Nagelkerke R Square is 0.745;

**-2 Log likelihood is 260.598, Cox & Snell R Square is 0.473, and Nagelkerke R Square is 0.688;

***-2 Log likelihood is 142.552, Cox & Snell R Square is 0.437, and Nagelkerke R Square is 0.719;

^#^Reference group is the very poor quintile.

Table 4 illustrates the protection against impoverishment and CHE provided by the NCMS. After healthcare expenditure, poverty prevalence increased markedly in all three provinces. After NCMS reimbursement, however, the prevalence of poverty and CHE decreased only slightly compared with the position after healthcare expenditure, although the change was more obvious in households with people suffering chronic diseases than those without. After healthcare expenditure and NCMS reimbursement, there were significant differences of prevalence of poverty and CHE between households with and without people suffering from chronic diseases.

**Table 4:** NCMS protection against impoverishment owing to health expenditure and CHE

	**Hubei**			**Chongqing**			**Zhejiang**		
	**Households with members suffering from chronic diseases**	**Households without chronic diseases**	p-value*	**Households with members suffering from chronic diseases**	**Households without chronic diseases**	p-value*	**Households with members suffering from chronic diseases**	**Households without chronic diseases**	p-value*
Poverty prevalence (N (%))									
Pre-payment	72 (24.57%)	56 (16.47%)	p = 0.011	46 (18.40%)	34 (13.71%)	p = 0.154	15 (10.49%)	19 (7.57%)	p = 0.321
Post-payment	158 (53.92%)	82 (24.12%)	p < 0.001	115 (46.00%)	52 (20.97%)	p < 0.001	51 (35.66%)	29 (11.55%)	p < 0.001
Post-reimbursement	148 (50.51%)	79 (23.24%)	p < 0.001	107 (42.80%)	51 (20.56%)	p < 0.001	44 (30.77%)	28 (11.16%)	p < 0.001
CHE prevalence (N (%))									
Post-payment	172 (58.70%)	52 (15.29%)	p < 0.001	116 (46.40%)	35 (14.11%)	p < 0.001	66 (46.15%)	23 (9.16%)	p < 0.001
Post-reimbursement	153 (52.22%)	44 (12.94%)	p < 0.001	106 (42.40%)	28 (11.29%)	p < 0.001	53 (37.06%)	17 (6.77%)	p < 0.001

*α = 0.05.

## Discussion

The primary purpose of any insurance system is to share risk between individuals or to smooth risks for an individual at different times, and so extend financial protection to the members. According to our analysis, NCMS does reduce the prevalence of poverty and CHE, at least in part, by decreasing medical expenditure, and provides additional protection to households with members suffering from chronic diseases. However, the financial protection afforded by NCMS is not strong enough to prevent continuing high levels of impoverishment and CHE in households with chronic diseases. For example, the prevalence of CHE after reimbursement is still over 50% in Hubei. This shows that households in rural China still have a high risk of a heavy financial burden from disease, even if they are covered by health insurance.

Although Wang et al. worried that the financial burden of chronic diseases would increase as China’s population aged [7], our logistic regression analysis demonstrates that the proportion of households with elder members has no direct relationship with CHE. Paez et al. [8] and Hwang et al. [19] also found that OOP payments and the prevalence of chronic diseases were increasing across the whole population, and not only among elder people, irrespective of sex, race, ethnicity, or income. Despite industrialized countries, especially Europe and Japan, having higher levels of chronic diseases than China, often ascribed to the higher percentages of older people in these countries [20], chronic disease comprises a growing share of the disease burden in developing countries [21,22].

Comparing the logistic regression equations for the three provinces reveals that CHE risk is associated with number of household members with chronic disease, and their utilization of medical treatment and healthcare expenditure. Hwang et al. also supported this conclusion, and revealed that the nearly linear relationship between OOP payments and chronic diseases persisted even after controlling for insurance status and other demographic factors [19]. Blendon et al. mentioned that despite substantial efforts to publicly fund essential care and support, OOP payments were financially stressful for people with chronic disease and their families [23].

One of the main reasons for high OOP payments in rural China is the limited drug and treatment catalogues of the NCMS, and the narrow gap between deductible and the reimbursement ceiling. In particular, high deductible for outpatient services creates a financial burden for households containing people with chronic diseases. Although the NCMS nominal reimbursement rate, the reimbursement ratio of service within the insurance drug and treatment catalogues in the designated medical institutions, has been gradually raised in recent years, and is now 95% for inpatient and 50% for outpatient services in township hospitals, the effective reimbursement ratio of both outpatient and inpatient services is far lower than the nominal reimbursement ratio, because of the high deductible, the low reimbursement ceiling, as well as expenditure on healthcare not included in the insurance catalogues. The NCMS therefore requires continuous review to enhance the effective reimbursement level for those who require high levels of assistance.

Considering that the result of the logistic regression showed an association between CHE risk and utilization of medical treatment, we conjectured that another reason for high OOP payments of people with chronic diseases could be health providers’ and patients’ behaviors. Health providers were motivated to provide clinical treatment rather than disease prevention and health education, or urged to provide more services than strictly necessary, because fee-for-service payment for providers still dominates in NCMS. Overuse of clinical services could also result from patients’ behavior, because it is difficult to judge rationality of utilization by rural residents. Unnecessary or unreasonable utilization of medical treatment not only increases OOP payments, but also increases the risk of health insurance fund. The role of health providers in medical care utilization is likely to be more important than that of patients, and health providers’ behavior should therefore be strictly regulated. Yip et al. [24] and Mechanic et al. [25] suggested that it might be possible by introducing the global budget and capitation payment pattern of health insurance to regulate the behavior of health providers, which could be a means to indirectly reduce patients’ financial burden.

Our study has several limitations. First, it may lead to recall bias for respondents to estimate healthcare expenditure over the year prior to the survey, especially for those with chronic disease. However, previous studies have suggested that recall on catastrophic health expenditure and impoverishment due to health expenditure barely fades over time [2]. Second, indirect health expenditure data were unavailable, so the true financial costs of obtaining healthcare may be underestimated. However, since only direct medical costs can be reimbursed directly by the NCMS, focusing on these direct costs matched our main interest.

## Conclusions

Modification of NCMS policies has provided more financial protection to households containing someone suffering from chronic diseases in rural China. However, these households continue to face a high financial risk, because the likelihood of impoverishment and CHE was still higher than for households without anyone suffering from chronic diseases. In view of the association between OOP and CHE risk and its possible reasons, we suggest that the NCMS should further improve its protection by broadening the catalogue of reimbursable drugs and treatments, decreasing or abolishing deductible and increasing the effective reimbursement ratio of outpatient service for people with chronic diseases, and with an accompanying expansion of NCMS fund. Health providers’ behavior should be modified by payment reform, which would not only assist the transformation of the health service model in rural China from treatment to preventive and rehabilitative care, especially for those with chronic diseases, but also better control health expenditure.

## Abbreviations

OOP: Out-of-pocket; CHE: Catastrophic health expenditure; NHSS: National Health Service Survey; NCMS: New Cooperative Medical Scheme.

## Competing interests

The authors declare that they have no competing interests.

## Authors’ contributions

JW and JM designed, planned and carried out the research and field survey. ZZ developed the methods for measuring and analyzing the results. JW and LC analyzed the data and drafted the initial manuscript. TY participated in the analysis of the results and helped modify various details of the article to produce a final version. All authors read and approved the final manuscript.

## Pre-publication history

The pre-publication history for this paper can be accessed here:

http://www.biomedcentral.com/1472-6963/14/305/prepub
